# Response to Stress in Early Tumor Colonization Modulates Switching of CD133-Positive and CD133-Negative Subpopulations in a Human Metastatic Colon Cancer Cell Line, SW620

**DOI:** 10.1371/journal.pone.0061133

**Published:** 2013-04-05

**Authors:** Chih-Sin Hsu, Chien-Yi Tung, Chih-Yung Yang, Chi-Hung Lin

**Affiliations:** 1 Institute of Microbiology and Immunology, National Yang-Ming University, Taipei, Taiwan; 2 VGH Yang-Ming Genome Research Center, Taipei, Taiwan; 3 Department of Education and Research, Taipei City Hospital, Taipei, Taiwan; Vanderbilt University Medical Center, United States of America

## Abstract

According to the cancer stem cell (CSC) model, higher CD133 expression in tumor tissue is associated with metastasis and poor prognosis in colon cancer. As such, the CD133-positive (CD133^+^) subpopulation of cancer cells is believed to play a central role in tumor development and metastatic progression. Although CD133^+^ cells are believed to display more CSC-like behavior and be solely responsible for tumor colonization, recent research indicates that CD133^−^ cells from metastatic colon tumors not only also possess colonization capacity but also promote the growth of larger tumors in a mouse model than CD133^+^ cells, suggesting that an alternative mechanism of metastasis exists. This study investigated this possibility by examining the cell viability, tumorigenicity, and proliferation and growth capacity of the CD133^+^ and CD133^−^ subpopulations of the SW620 cell line, a human metastatic colon cancer cell line, in both an *in vitro* cell model and an *in vivo* mouse model. While both SW620 ^CD133−^ and SW620^CD133+^ cells were found to engage in bidirectional cell-type switching in reaction to exposure to environmental stressors, including hypoxia, a cell adhesion-free environment, and extracellular matrix stimulation, both *in vitro* and *in vivo*, CD133^−^ cells were found to have a growth advantage during early colonization due to their greater resistance to proliferation inhibition. Based on these findings, a hypothetical model in which colon cancer cells engage in cell-type switching in reaction to exposure to environmental stressors is proposed. Such switching may provide a survival advantage during early colonization, as well as that explain previous conflicting observations.

## Introduction

Most deaths due to colon cancer are correlated with metastasis of the primary tumor rather than development of the primary tumor. As such, gaining understanding of colon cancer metastasis could greatly increase the patient survival rate. Achieving such a goal is particularly important, as the median overall survival duration of metastatic colon cancer patients is currently less than 2 years and the 5-year survival rate less than 10% [1], [2]. Gaining understanding of metastasis could also greatly assist in selecting the appropriate therapy strategy.

It is currently known that tumor metastasis is a multi-stage process in which the malignancy of tumor cells progressively develops in 3 sequential stages: intravasation, extravasation, and colonization [3]. In the intravasation stage, during which tumor cells undergo cellular functional changes in preparation for cell migration and extracellular matrix (ECM) digestion, tumor cells begin to escape from the primary site and invade the circulating system. In the extravasation stage, tumor cells circulate in blood vessels and the lymphatic system before being transported to distant metastatic sites. During the cancer colonization stage, the transported tumor cells adapt to the microenvironment and begin to proliferate. In the early colonization stage, tumor cells are challenged by many environmental stressors, such as ECM interactions, hypoxic conditions, growth-factor stimulation, and immune-system interactions, that lead to their death or dormancy [3]. The few cells that can adapt to the stressors and survive become the subpopulation of cancer cells involved in cancer colonization and are responsible for the metastasis procession.

Recently, a model of cancer stem cells (CSCs) has been proposed and widely discussed [4]. This model is based on previous research in which a small subpopulation of cancer cells isolated from the primary tumor that are characterized by high apoptosis resistance, long lifespan, and high differentiation and clonal expansion capability were defined as cancer stem cells [5]. According to the CSC model, all tumor cells released from the primary site are disseminated, but only CSCs can survive and establish colonies at metastatic sites. Several studies have reported that CSCs participate in metastasis progression [4], [6]. A number of cell markers, including CD133, CD166, and CD26, have been used to isolate the CSC subpopulation within colon-cancer-cell populations [7], [8]. Among them, prominin 1 (CD133) is a pentaspan transmembrane protein originally found in hematopoietic stem cells in the bone marrow [9]. Recent studies have reported CD133 to be a potential biomarker of metastasis and poor prognosis in colon cancer patients [10]–[12]. In accordance, immunohistochemical (IHC) staining of colon cancer tissue has revealed high expression of CD133 to be correlated with liver metastasis and low survival [12], [13].

The CD133 protein has been found to be more abundant in liver metastatic lesions compared with primary colon cancer lesions [14]. Nevertheless, CD133 is widely considered a CSC marker in colon cancer. As downregulation of CD133 has been widely observed during tumor development, its expression is believed to be regulated during cell differentiation [15]. In a mouse xenograft experiment investigating the CD133-negative (CD133^−^) and CD133-positive (CD133^+^) subpopulations of primary colon cancer cells, only the CD133^+^ subpopulation was found capable of inducing tumor formation [15]–[17]. These findings, in addition to the wide and effective use of CD133 as a biomarker to isolate stem cells in cancerous tissues [18], [19], suggest that a subpopulation of CD133^+^ cells acts as CSCs in the metastasis of colon cancer.

However, several controversial findings have emerged from comparison of CD133^−^ and CD133^+^ subpopulations in cell-lines and primary cultures of colon cancer [20]–[23]. Although both CD133^−^ and CD133^+^ subpopulations of HCT-116 cells, a human colon cancer cell line, have been found to induce tumor growth in mouse models [22], the CD133^−^ subpopulation of one human metastatic colon cancer cell line, LoVo, was found more resistant to 5-fluorouracil treatment than the CD133^+^ subpopulation [20]. Likewise, Shmelkov et al. found a subpopulation of CD133^−^ colon cancer cells isolated from a liver metastatic site in a xenograft mouse model to have a higher level of tumorigenicity than the subpopulation of CD133^+^ cells [23]. These conflicting results cannot be explained by the CSC model, and suggest the existence of an alternative mechanism in metastasis procession.

SW620 is a human colon-cancer cell line that originates from a lymph node metastatic site. As it possesses both CD133^+^ and CD133^−^ subpopulations in a general culture condition (i.e., that obtained in a dish containing an L15 medium and 10% FBS at 37°C), SW620 can serve as an excellent cell line model with which to investigate the differences between CD133^+^ and CD133^−^ subpopulations within cells derived from the same genetic background. Using this model, this study isolated the CD133^+^ and CD133^−^ subpopulations in a SW620 cell line to compare their tumorigenicity, cell behavior, and proliferation and growth capacity both *in vitro* and *in vivo*. Based on the results, a hypothetical model of cancer colonization in metastasis that explains the controversial findings of recent studies is proposed.

## Results

### The SW620 cell line consists of 2 subpopulations with high and low CD133 expression

**Figure 1:**
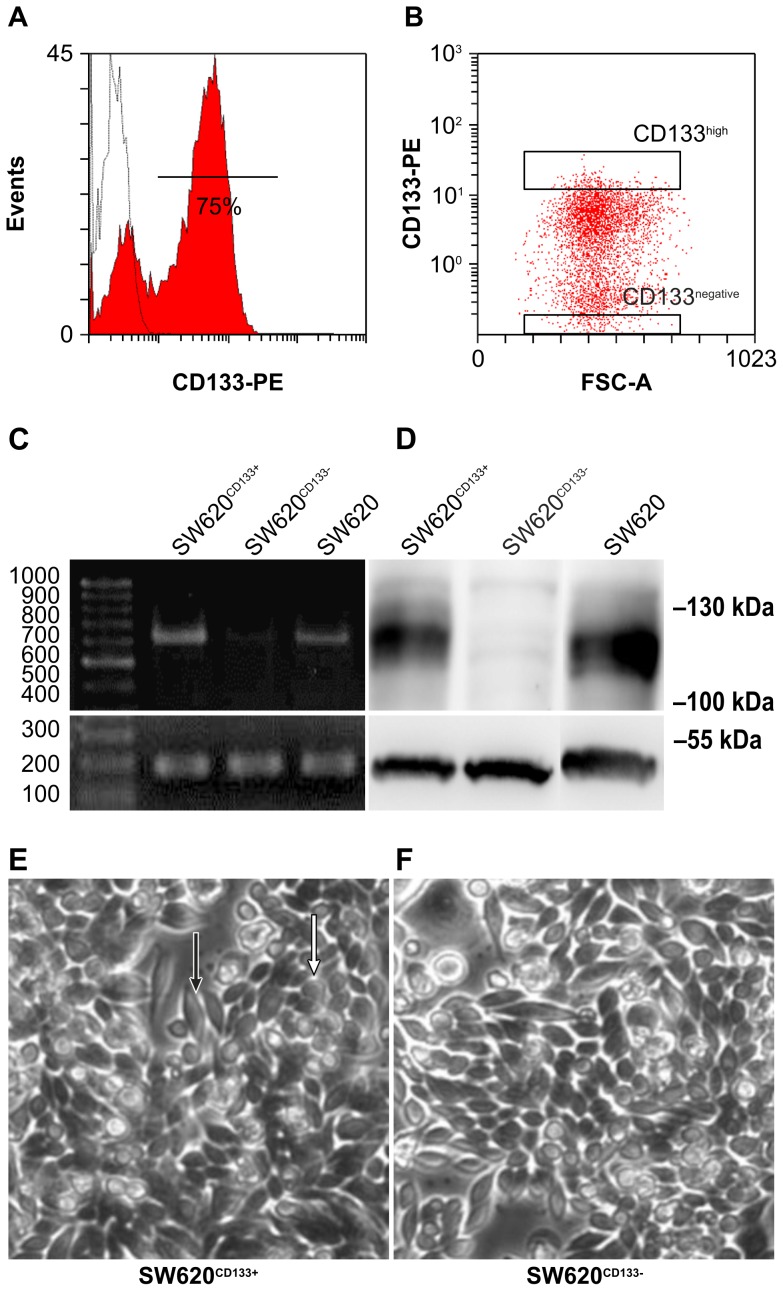
Purification of SW620^CD133+^ and SW620^CD133−^ cells. The CD133 expression of SW620 cells was analyzed and the cells were sorted by FACS Canto and FACS Aria, respectively. (A) Measurement of CD133 expression by flow cytometry revealed that 75% of the cells were SW620^CD133+^ cells and 25% were SW620^CD133−^ cells (white peak indicates staining control for mouse IgG). (B) The top 5% of the CD133+ subpopulation (CD133^high^) and bottom 5% of the CD133− subpopulation (CD133^negative^) were collected using FACS Aria. (C) Upper panel shows RT-PCR results for CD133 mRNA. Lower panel shows RT-PCR results for GAPDH mRNA used as a control to perform normalization. (D) Upper panel shows western blot results for CD133 protein in total cell lyse. Lower panel shows the results for alpha tubulin used as a loading control. (E) SW620^CD133+^ cell morphology under light microscopy (20×). (F) SW620^CD133−^ cell morphology under light microscopy (20×).

Figure 1A shows that the SW620 cell line was divided into 2 populations, the SW620^CD133+^ and SW620^CD133−^ subpopulations, according to CD133-expression level, of which the SW620^CD133+^ population occupied 75% of the SW620 cells in a general culture condition. To isolate the subpopulations, fluorescent-activated cell sorting and analysis (FACS) was performed and subsequently confirmed by reverse-transcription polymerase chain reaction (RT-PCR) and western blot. The highly and poorly expressed subpopulations were then identified by performing analysis of CD133^high^ and CD133^negative^ zone distribution (Figure 1B). The results revealed that the SW620^CD133+^ cells express more CD133 mRNA and protein than SW620^CD133−^ cells (Figure 1C and 1D), which excluded the possibility of alternative splicing or translocation regulation [24]. It is well known that SW620 contains 2 distinct morphological cell types, a small spherical (white arrow, Figure 1E; Figure S1A and S1B) and a bipolar type (black arrow, Figure 1E; arrow-head, Figure S1A and S1B) [25], whose proportion does not significantly differ in the SW620^CD133+^ and SW620^CD133−^ subpopulations.

Carboxyfluorescein diacetate–succinimidyl ester (CFSE) staining of FACS and MTT assay revealed that the proliferation ability between SW620 subpopulations are similar in a general culture condition (Figure S1C and S1D), while MTT assay with taxol, cisplatin, actinomycin D, and camptothecin treatment revealed the cell viability of the subpopulations to be similar (Figure S2A to S2D). These results indicate that SW620^CD133+^ and SW620^CD133−^ cells have no apparent morphological and physiological differences in a general culture condition. As these results cannot explain the different levels of tumorigenicity between the CD133 subpopulations found in previous studies [23], they suggest that inducement of different levels of tumorigenicity can be attributed to exposure to particular environmental conditions.

### SW620^CD133−^ cells exhibit higher colony formation capacity than SW620^CD133+^ cells in a 3D Matrigel culture

**Figure 2:**
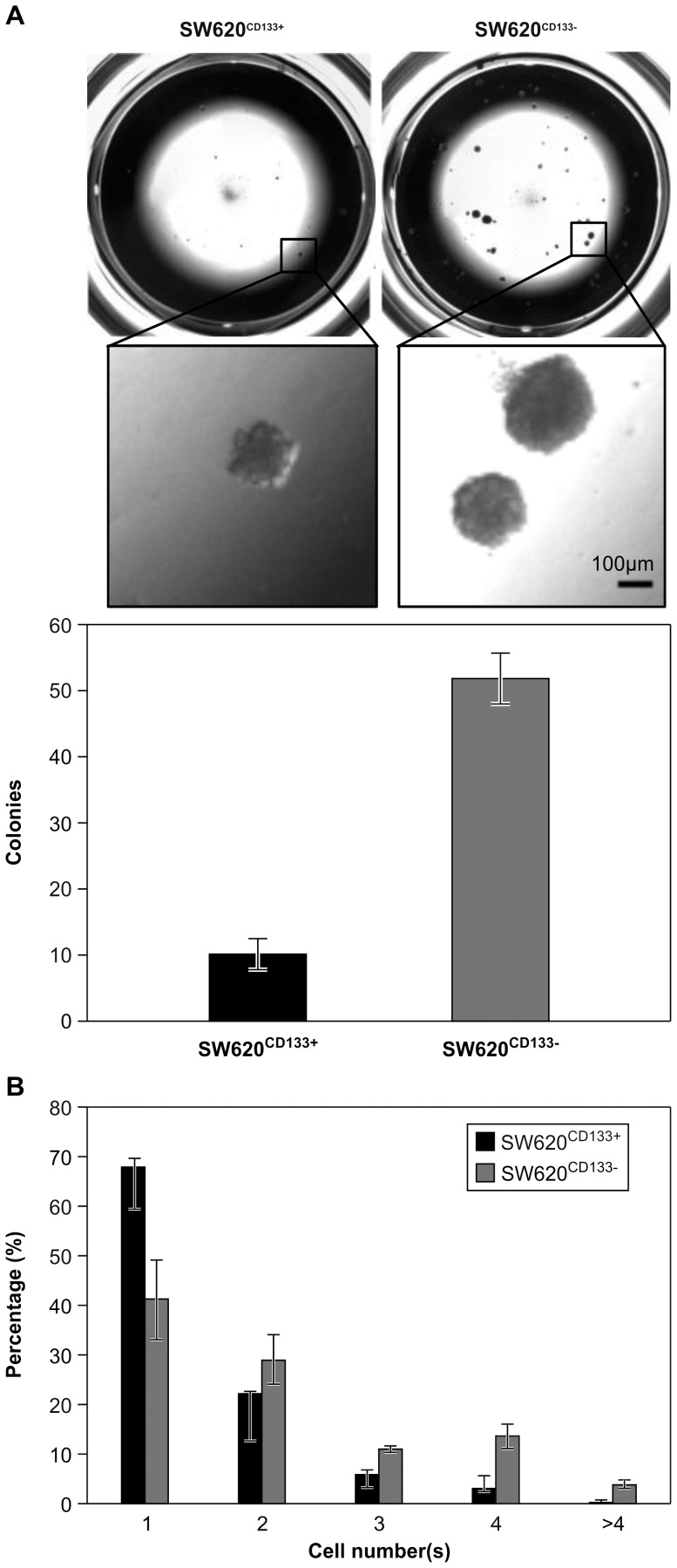
Comparison of colony formation capacity of SW620^CD133+^ and SW620^CD133−^ cells on 3D Matrigel culture. Purified SW620^CD133+^ and SW620^CD133−^ cells were seeded on top of Matrigel for further analysis of cell proliferation and colonization. (A) Upper panel shows the entire and enlarged picture for colonies morphology; lower panel shows colonies count after 3 weeks of incubation. The SW620^CD133+^ cells formed a mean of 10 colonies (SD = 2.2) and the SW620^CD133−^ cells a mean of 50 colonies (SD = 3.8). Bar = 100 µm. (B) Cell count after 3 days of incubation.

The ability of extravasated tumor cells to grow despite immediate exposure to microenvironmental stressors at the metastatic site is essential for metastatic cells to progress to tumor colonization [26]. Matrigel, a soluble basement membrane protein extract derived from the Engelbreth-Holm-Swarm mouse sarcoma, can serve as an *ex vivo* microenvironment in which cells are exposed to 3D architecture, ECM interaction, and growth factor stimulation [27]. As such, a 3D Matrigel culture model has become widely used as an experimental model to measure tumorigenicity [28]–[30]. Using this model to compare the colony formation capacity of SW620^CD133+^ and SW620^CD133−^ cells, 500 SW620^CD133+^ and SW620^CD133−^ cells were seeded on top of a thick Matrigel culture. Quantification of visible colonies after 3 weeks of culture revealed that the SW620^CD133−^ cells, which had formed a mean of 51.8 colonies (SD = 3.8), had a higher colony formation capability compared to the SW620^CD133+^ cells, which had formed a mean of 10.3 colonies (SD = 2.2; Figure 2A).

Cell proliferation in the early phase was further examined by quantification of the cell number in each clone. When approximately 200 cells were seeded in a low-cell-density on 3D Matrigel culture and the cell number of each colony was counted one by one under microscope after 3 days incubation, the results revealed that the SW620^CD133+^ cells were less proliferative than SW620^CD133−^ cells (Figure 2B). Specifically, only a small proportion of SW620^CD133+^ cells (black bar) had been able to complete 2 (cell number = 4, 3.4%) or more (cell number >4, 0.4%) rounds of cell cycle, and most had experienced arrested growth, such that only 22.5% divided one time to become 2 cells and 67.7% did not undergo any cell division at all. The SW620^CD133−^ cells (gray bar) were found to be more tolerant to proliferation inhibition due to exposure to the 3D Matrigel culture, with 4.7-fold more SW620^CD133−^ cells (18%) than SW620^CD133+^ cells having been able to complete ≥2 rounds of cell cycle. This 4.7-fold difference in large colony percentage (n≥4) between the 2-cell types is similar to the 5-fold difference in the number of visible colony numbers previously observed. This result indicates that different levels of tolerance, specifically the higher level of SW620^CD133−^ cells, to proliferation inhibition from the microenvironment leads to different levels of tumorigenicity between SW620^CD133+^ and SW620^CD133−^ cells, providing SW620^CD133−^ cells with greater capacity to engage in tumor formation at early metastatic sites.

### SW620^CD133−^ cells have a higher level of tumorigenicity than SW620^CD133+^ in a nude mouse model

**Figure 3:**
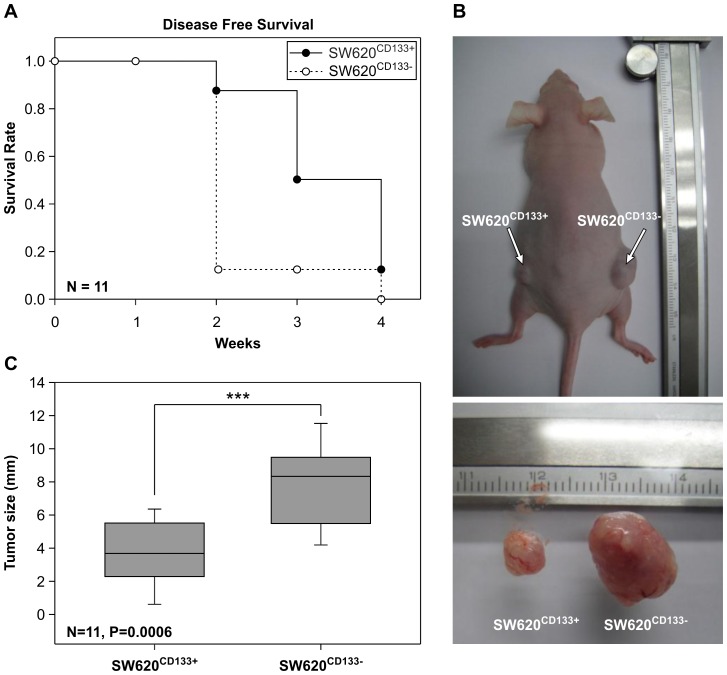
Comparison of tumorigenicity of SW620^CD133+^ and SW620^CD133−^ cells in nude mice. Purified SW620^CD133+^ and SW620^CD133−^ cells were injected subcutaneously into nude mice. (A) Filled and unfilled circles refer to the disease-free survival rate of the SW620^CD133+^ and SW620^CD133−^ cells, respectively, N = 11. (B) Upper photograph shows tumor formation due to SW620^CD133+^ cell inoculation on the left side and due to SW620^CD133−^ cell inoculation on the right side. Lower photograph shows the sizes of the SW620^CD133+^ and SW620^CD133−^ tumors. (C) Chart showing the mean diameters of the SW620^CD133+^ (3.8 mm, SD = 2.1) and SW620^CD133−^ (8.1 mm, SD = 2.6) tumors, N = 11, P = 0.0006.

The *in vivo* tumorigenicity of SW620^CD133+^ and SW620^CD133−^ cells was examined in a nude (nu/nu) mouse model. After inoculation of 10^5^ cells in a subcutaneous region, tumor size was measured every 3 days for 4 weeks. The disease-free survival rate is shown in Figure 3A. Both cell types were able to generate tumor tissue after 4 weeks inoculation. Although hematoxylin and eosin (H&E) staining revealed the tumor morphology of the 2 cell types to be similar (Figure S3A and S3B), the growth rate was found to differ, with SW620^CD133−^ cells found able to generate larger tumor cells at an earlier stage. As shown in Figure 3C, by 2 weeks post inoculation, 78% of the mice inoculated with SW620^CD133−^ cells had developed tumors >2 mm in size (open circle) while only 11% of the mice inoculated with SW620^CD133+^ cells had done so (filled circle). Moreover, the average diameter of the tumors that had developed after SW620^CD133−^ inoculation was 1.9-fold greater than that after SW620^CD133+^ inoculation (Figure 3B and 3C). These results, which confirm that SW620^CD133−^ cells have greater tumorigenicity than SW620^CD133+^ cells and accord with the results obtained using the *ex vivo* experimental model, provide the first cell-line evidence that the CD133^−^ subpopulation has greater potential to initiate tumor progression at metastatic sites [23].

### Cell-type switching between SW620^CD133−^ and SW620^CD133+^ subpopulations is induced by microenvironmental stimulation

Although inducement of CD133 upregulation by exposure to certain stressors and forms of environmental stimulation was observed in several previous *in vitro* cell experiments [31]–[33], switching of the CD133^−^ subpopulation to the CD133^+^ subpopulation of brain tumor primary cultured cells was observed in a recent *in vivo* experiment [34]. Similar switching was observed in the NANK cell line, a primary colon cancer line. Although NANK is a CD133^−^ cell line isolated from ovarian metastatic colon cancer tissues, CD133^+^ cells were found in a NANK-induced tumor [21].

**Figure 4:**
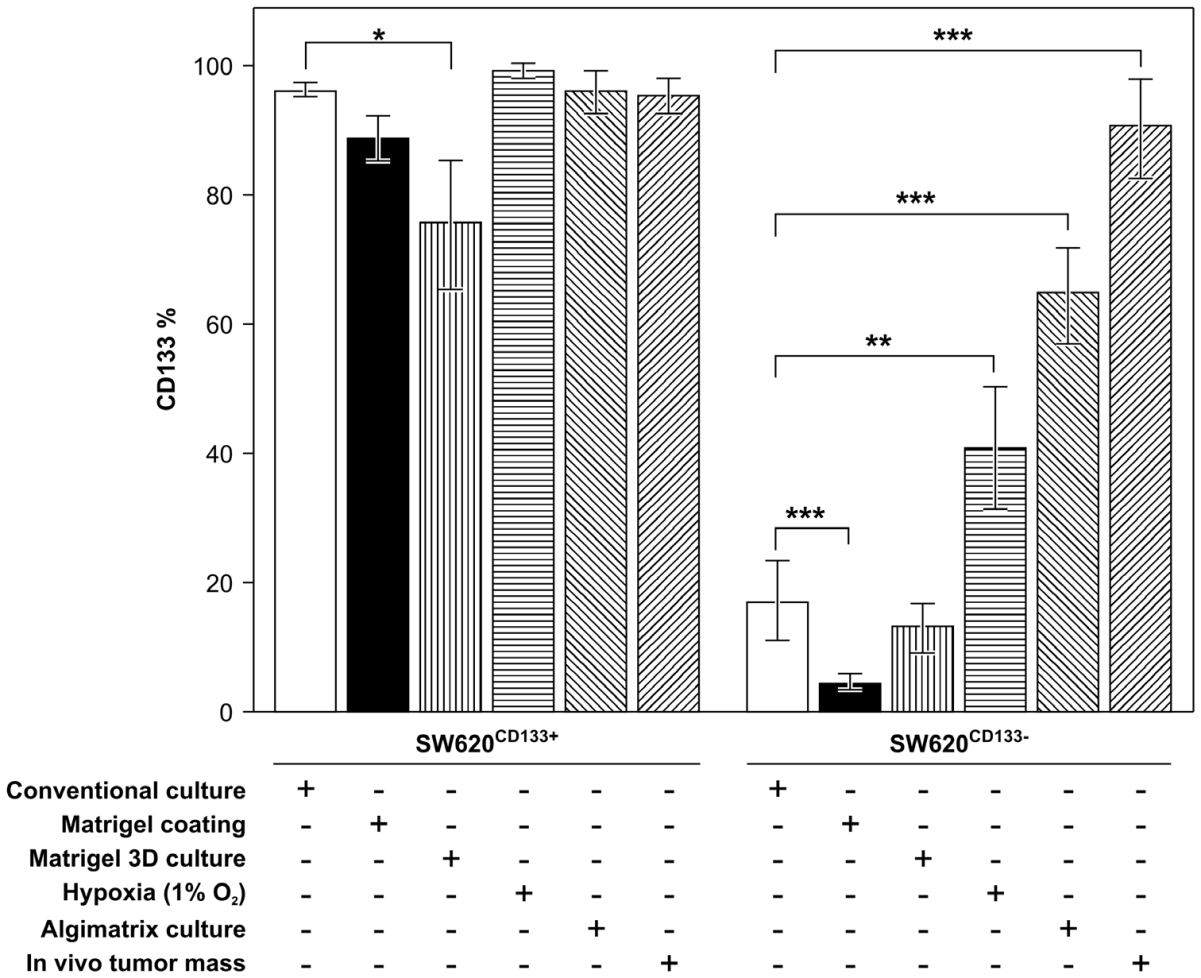
Modulation of CD133 expression in SW620 cells in reaction to exposure to stressors. Left panel shows SW620^CD133+^ cells and right panel shows SW620^CD133−^ cells. Detail descriptions were shown in result.

In accordance with these findings, switching between SW620^CD133−^ and SW620^CD133+^ cells in an unbalanced manner was observed in the general culture condition in this study. Specifically, 16.7% of purified SW620^CD133−^ cells switched to SW620^CD133+^ cells (Figure 4, white bar in right panel) while only 4.7% of SW620^CD133+^ cells switched to SW620^CD133−^ cells (white bar in left panel, Figure 4). This 3.6-fold difference in switching capacity between SW620^CD133−^ and SW620^CD133+^ cells may explain the greater proportion of SW620^CD133+^ compared to SW620^CD133−^ cells (3∶1) in the general culture condition (Figure 1). This explanation is supported by the results of the MTT assay, which revealed that the 2 populations have a similar proliferation rate, and thus excluded the possibility of growth competition.

Investigation of the tumor cells isolated during the previous xenograft experiments (Figure 3A) confirmed these findings. Whereas 90.3% of tumor cells that had proliferated from SW620^CD133−^ cells later switched to SW620^CD133+^ cells, only 4.8% of SW620^CD133+^ cells switched to SW620^CD133−^ cells (Figure 4, diagonal bar). These results suggest that cell-type switching might be modulated by stimulation from the *in vivo* microenvironment. To test this hypothesis, the impact of exposure to hypoxia, a cell-adhesion-free environment, and ECM stimulation, 3 potential forms of stress in the *in vivo* microenvironment was examined. To establish a hypoxic condition, the oxygen concentration in the culture chamber was controlled at 1% using the PROOX 110 instrument and the temperature maintained at 37°C. Whereas exposure to hypoxia significantly increased switching of SW620^CD133−^ cells to SW620^CD133+^ cells, specifically from 16.7% to 40% (p = 0.0064), it resulted in no significant change in the extent to which SW620^CD133+^ cells switched to SW620^CD133−^ cells (p = 0.13) after 4 weeks of culture in the hypoxia condition.

To test exposure to a cell-adhesion-free condition using a 3D culture system, cells were loaded onto Algimatrix, an artificial bio-scaffold material without an ECM component. Cells were cultured at low density (10^5^ per well) to prevent cell–cell contact. After 2 weeks of culture, cells were isolated from the gel and CD133 expression was measured by FACS. Whereas exposure to a cell-adhesion-free environment significantly increased the switching of SW620^CD133−^ cells to SW620^CD133+^, specifically from 16.7% to 64.5% (p<0.001), it resulted in no significant change in the extent to which SW620^CD133+^ cells switched to SW620^CD133−^ cells (p = 0.57). To examine the impact of ECM stimulation, cell culturing was performed in a Matrigel-coated plate. Although exposure to ECM stimulation significantly inhibited CD133 expression in both cell types, it had a variable impact on the extent of cell-type switching. Whereas it decreased switching of SW620^CD133−^ cells to SW620^CD133+^ cells from 16.7% to 5% (black bar in right panel, p<0.001) in a Matrigel-coated plate and to 13% in a 3D Matrigel culture (Figure 4, right panel, horizontal filled bar, p = 0.19), it increased switching of SW620^CD133+^ cell group from 5% to 11% in a Matrigel-coated plate (p = 0.055) and to 27% in a 3D Matrigel culture (Figure 4, left panel, horizontal filled bar, p = 0.023).

Testing of exposure to the 3 forms of environmental stress revealed the existence of a significant regulation of CD133 switching in response to such exposures, with exposure to hypoxia and a cell-adhesion-free environment significantly promoting switching of SW620^CD133−^ cells to SW620^CD133+^ cells while exposure to ECM stimulation significantly promoting switching of SW620^CD133+^ cells to SW620^CD133−^ cells. These results suggest that regulation of SW620^CD133−^ and SW620^CD133+^ subpopulations in an *in vivo* environment is modulated by exposure to multiple factors that act synergistically upon the cells, and that cell-type switching between subpopulations might be required for adaptation to the microenvironment in early tumor colonization.

## Discussion

It has been widely discussed that CSCs play a key role in metastasis progression [7], [35]. Of the several cell markers, including CD133, CD144, and CD166, that have been used to identify CSCs, a number of independent studies have demonstrated that the CD133^+^ subpopulation of colon cancer cells possess higher levels of stemness and tumorigenicity that the CD133^−^ subpopulation. Two independent studies that isolated CD133^+^ and CD133^−^ subpopulations from colon tumor cells and transplanted them into NOD/SCID mice found that only CD133^+^ cells can initiate tumor formation [15], [17]. A study using a xenograft model found that the CD133^+^ subpopulation of colon tumor cells also display multi-lineage differentiation capacity and a high level of tumorigenicity [16].

However, 2 recent studies found that the CD133^−^ subpopulation of metastatic colon cancer cells in liver and ovarian cancer also initiate tumor formation, and ultimately induce greater tumor growth than the CD133^+^ subpopulation [21], [23]. The conflicting nature of these various results may be due to the use of experimental models derived from different genetic backgrounds. The colon cancer cell line model provides a conventional and reproducible experimental model that allows for comparison of the 2 CD133 subpopulations. Of the 2 cell lines used as experimental models of colon cancer that can be divided into CD133^+^ and CD133^−^ subpopulations [32], the HCT116 cell line, which was established primarily from colon tumors, has a smaller (<5%) CD133^−^ subpopulation than the SW620 cell line. In accordance with previous comparisons of cell viability and tumorigenicity between HCT116^CD133−^ and HCT116^CD133+^ cells by MTT assay and xenograft experiments, HCT116^CD133−^ and HCT116^CD133+^ cells have been found to have similar levels of viability and tumorigenicity [22].

In the present study, the SW620^CD133−^ and SW620^CD133+^ subpopulations were found to have a similar morphology, proliferation rate, and drug response in a general culture condition. However, the SW620^CD133−^ cells displayed significantly greater growth in a 3D Matrigel culture and in mouse xenograft experiments. These results provide the first pieces of evidence that CD133^−^ colon cells have greater tumorigenicity than CD133^+^ colon cells in experimental models with the same genetic background, suggesting that certain environmental factors could induce different rates of growth. Unfortunately, exposure to one form of stress, such as hypoxia or ECM stimulation, alone, was not found to affect the proliferation rate (Figure S4A), indicating that inhibition might be induced by a complex mechanism of regulation and/or a number of factors that act synergistically in the microenvironment. The existence of such a complex mechanism remains to be investigated.

Switching between CD133^+^ and CD133^−^ subpopulations has been observed in many types of tumor cells [36]–[38]. It is well known that CD133^+^ CSCs will switch to CD133^−^ cells during differentiation [16], and that such switching is unidirectional and irreversible. In the chemically induced differentiation model, sodium butyrate treatment of the human colon cancer cell lines HT-29 and HCT116 has been found to induce CD133 downregulation and epithelial differentiation [39]. Several tumor studies have found that switching of CD133^−^ cells to CD133^+^ cells can be induced by exposure to growth factor and environmental stressors. Specifically, exposure to TGF-beta1 has been found to trigger CD133 upregulation in the Huh7 and A549 hepatocellular carcinoma and lung cancer cell lines and increase cell tumorigenicity in a nude mice model [33], [36]. While exposure to hypoxia has been found to induce CD133 expression in lung and pancreatic cancer cells, these transformed CD133^+^ tumor cells gain more stemness and malignancy [31], [40]. Hypoxia induced CD133 expression in brain tumor also correlates with higher tumor aggressiveness [41].

In a xenograft model of metastatic colon tumor, CD133^+^ cells were detected in a tumor generated from purified CD133^−^ cells [21], suggesting that cell-type switching might also occur in colon cancer cells. The results of this study clearly demonstrate the existence of bi-directional switching between SW620^CD133+^ and SW620^CD133−^ subpopulations, and that such switching can be modulated by exposure to various stressors. Specifically, exposure to either a Matrigel-coated plate or a 3D Matrigel culture was found to significantly induce switching of SW620^CD133+^ to SW620^CD133−^ cells, the latter of which has been found to exhibit greater growth in *ex vivo* and *in vivo* models. In pilot studies, exposure to Type I and IV collagen and laminin, the 3 major ECM components, was observed to induce inhibition of CD133 expression to a similar extent (data not shown). In contrast, exposure to hypoxia or a cell-adhesion-free condition was found to promote switching of SW620^CD133−^ cells in the present study. This result was also observed using the hand-drop culture system, which provides exposure to a cell-adhesion-free environment, in which SW620^CD133+^ cells were found in SW620^CD133−^-forming spheres, suggesting that they play a role in driving tumorigenesis [32].

**Figure 5:**
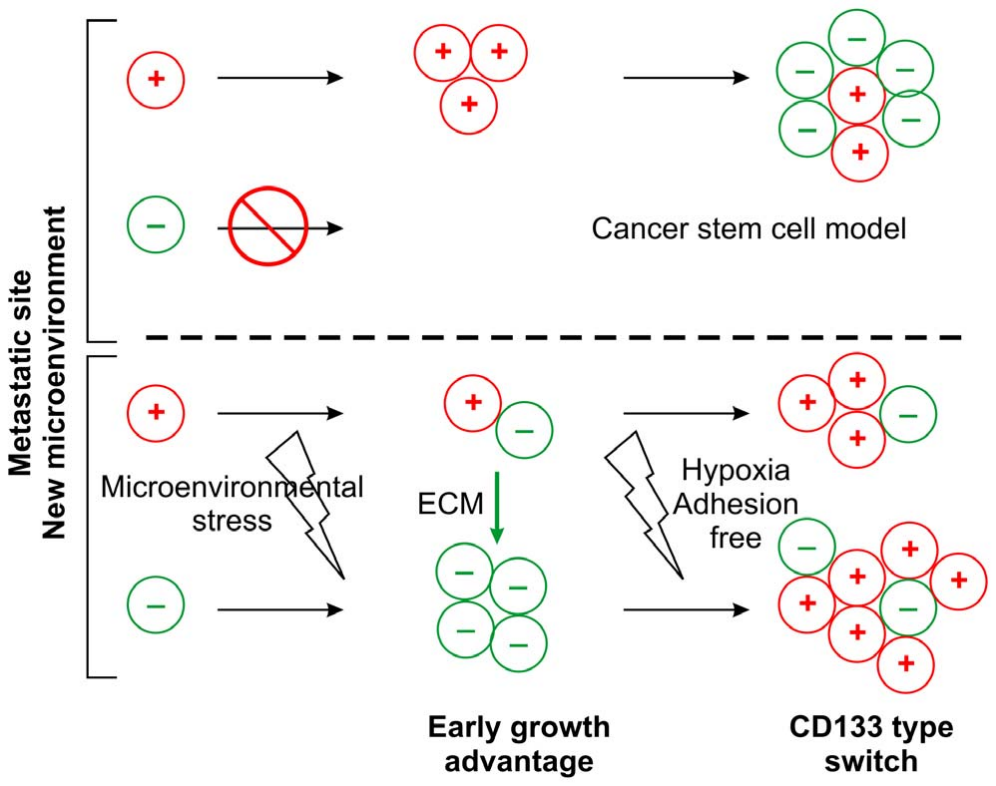
Hypothetical model of early tumor colonization in metastatic lesions. Upper panel shows conventional CSC model, in which only CD133^+^ colon cancer cells colonize at the metastatic site before differentiating into CD133^−^ cells during tumor growth. Lower panel shows the hypothetical model, in which both CD133^+^ and CD133^−^ cells colonize at the metastatic site and in which CD133^−^ cells have greater colonization capacity. While ECM stimulation promotes switching of CD133^+^ cells to CD133^−^ cells in the early phase of colonization, exposure to hypoxia and 3D architecture after colonization induces switching of CD133^−^ cells to CD133^+^ cells.

In the conventional model of metastasis progression, both CD133^+^ and CD133^−^ cancer cells are released into the bloodstream when metastasis is initiated [42], but only CD133^+^ cells are able to survive and initialize tumor colonization (Figure 5, upper panel). However, several studies of colon cancer cell lines have reported that both CD133^+^ and CD133^−^ cells can initialize tumor formation [21]–[23]. The present study, which observed this phenomenon in the SW620 cell line, also found that colon cancer cells may engage in cell-type switching between the SW620^CD133−^ and SW620^CD133+^ subpopulations in response to microenvironmental stress. Based on these observations, an alternative hypothetical model of colon cancer colonization at the metastasis site, which is illustrated in Figure 5, is proposed here. In the early metastatic phase, cancer cells migrate into the metastatic site and begin colonizing. During this phase, the cells are directly exposed to many forms of environmental stress and stimulation, among which ECM stimulation promotes the switching of CD133^+^ to CD133^−^ cells, the latter of which have a growth advantage due to their greater resistance to proliferation inhibition from the microenvironment. After tumor colonization has been established and the tumor cells have been organized into a solid tumor, other forms of environment stress, including exposure to hypoxia and a cell-adhesion-free environment, promote the switching of CD133^−^ to CD133^+^ cells, which, because the latter type has been found to have greater stemness and malignancy in colon and other types of cancer [36], [43], may increase later tumor growth.

This hypothetical model explains the conflicting results obtained from previous studies of CD133^+^ and CD133^−^ colon cancer cells. Although CD133 is known as a stem-cell marker, the results of previous studies, as well as the present study, indicate that both CD133^+^ and CD133^−^ cells can generate tumor growth, suggesting that CD133 might not be an absolute biomarker for cancer stem cells in colon cancer. To help cancer cells adapt to different environmental stressors during tumor regression, the 2 subpopulations often engage in cell-type switching, which may explain why CD133^+^ cells are frequently found at metastatic sites. This model provides a means of explaining the many observations regarding colon cancer metastasis that cannot be explained by the conventional CSC model.

## Materials and Methods

### Cell culture and growth conditions

The SW620 human colorectal cancer cell line used in this study was obtained from the Bioresource Collection and Research Center (Hsinchu, Taiwan). SW620 cells were grown in Leibovitz's L-15 Medium supplemented with 10% fetal bovine serum and 1% penicillin/streptomycin (Invitrogen) at 37°C. Cells were grown in a humidified environment to 80% confluence before further experiments and passage. A hypoxic condition consisting of 1% O_2_ and 99% N_2_ was controlled using the PROOX 110 instrument (BioSpherix, Lacona, NY, USA). Western blot for HIF-1alpha was performed to confirm achievement of a hypoxic condition. Matrigel was diluted with a serum-free medium (1∶20) and coated on a Petri dish to conduct the experiments that followed.

### FACS

After removal from the cell culture medium and washing in phosphate-buffered saline (PBS), cells were dissociated from the Petri dish using TE buffer (Invitrogen) and labeled with mouse anti-human CD133-PE antibody (Miltenyi Biotec) following the standard protocol on ice for 30 min. The labeled cells were then analyzed using FACS Canto (BD, San Jose, CA, USA) and sorted using FACS Aria (BD). The top 5% of the CD133^+^ subpopulation and bottom 5% of the CD133^−^ subpopulation were collected for further experiments.

### CFSE staining

For this process, 1×10^6^ cell/ml were labeled with 5 µM of CFSE (Invitrogen) at 37°C for 10 min, and 5×10^5^ cells were seeded onto a Petri dish for further culturing. Staining with CD133-APC antibody was performed on days 0 and 3 to analyze CD133 expression and cell division by FACS Canto.

### RT-PCR

The CD133 protein has been found to have various splice forms that can lead to loss of the AC133 epitope on the cell surface during cell differentiation but not of the CD133 protein [24]. Based on this finding, a specific primer was designed to amplify the conserved region for all CD133 alternative splicing forms on exons 17 to 24 in the CD133 gene using RT-PCR. Purified SW620^CD133+^ and SW620^CD133−^ cells were treated with TRIzol (Gibco-BRL; Gaithersburg, MD, USA) following the standard protocol to extract total RNA and reverse-transcribed into first-strand cDNA. Subsequently, cDNA was added to the PCR reaction with CD133 forward (5′- ACGGCACTCTTCACCTGCAG-3′) and reverse (5′-CGATGCCACTTTCTCACTGA-3′) primers (94°C for 30 s, 55°C for 30 s, and 72°C for 30 s for 27 cycles). Glyceraldehyde 3-phosphate dehydrogenase (GAPDH), forward (5′- AGCGAGATCCCTCC -3′) and reverse (5′- GCAGGAGGCATTGC -3′) was used as a control. The PCR product was separated on 2% agarose gel and the signal measured at 592 bp and 221 bp respectively.

### *In vivo* tumorigenicity assay

Six-to-eight week old immunodeficient nu/nu mice were obtained from BioLasco Taiwan Co. Ltd. and maintained within facilities operated by the National Yang-Ming University Laboratory Animal Center. Approval for all mouse experiments was obtained from the Institutional Animal Care and Use Committee of the National Yang-Ming University. Purified SW620^CD133+^ and SW620^CD133−^ cells (1×10^5^) were suspended in PBS and injected subcutaneously into the dorsal region near the thigh, which was then observed twice a week for 4 weeks. Mice were sacrificed 4 weeks after inoculation for excision of tumors and preparation of paraffin-embedded sections to H&E staining. Excised tumors were incubated with 10 U of collagenase I (Sigma-Aldrich) for 2 h at 37°C, washed twice with PBS, and filtered with a mesh to obtain single cells for CD133 expression analysis by FACS.

### Western blot analysis

Cells were washed twice with cold PBS and lysed by RIPA buffer (Cell Signaling Technology) with protease inhibitors (Roche) on ice. Equal amounts of proteins were separated by SDS-PAGE and transferred to polyvinylidene difluoride (PVDF) membranes. Membranes were blotted with anti-CD133 monoclonal antibody (Cell Signaling Technology), anti-HIF1alpha antibody (Cell Signaling Technology), anti-alpha tubulin antibody (Abcam), and secondary antibodies conjugated with horseradish peroxidase (HRP). The proteins were detected using an enhanced chemiluminescence kit (Pierce, Rockford, IL) in 133 kDa for CD133, 120 kDa for HIF1-alpha and 50 kDa for alpha tubulin.

### MTT assay

Purified SW620^CD133+^ and SW620^CD133−^ cells (1×10^3^) were seeded into 96-well plates and treated with different anti-tumor drugs, including taxol (Sigma-Aldrich, 1 uM), cisplatin (Sigma-Aldrich, 10 uM), actinomycin D (Millipore, 10 nM), and camptothecin (Millipore, 2 nM) to perform drug-resistance assay. After 24, 48, and 72 h of incubation, MTT reagent was added and the cells incubated at 37°C for 3 h. The solution was then replaced with DMSO to determine the absorbance at OD590 using a 620-nm reference filter.

### 3D Algimatrix culture

Purified SW620^CD133+^ and SW620^CD133−^ cells (1×10^5^) were seeded onto Algimatrix and cultured in a humidified environment at 37°C. After 2 weeks of incubation during which the medium was replaced every 3 days, the cells were dissociated from the Algimatrix using Algimatrix-dissolving buffer (Invitrogen), following the standard protocol to label with mouse anti-human CD133-PE antibody on ice for 30 min and analysis of CD133 expression by FACS.

### 3D Matrigel culture

In accordance with Lee et al.'s protocol [30], 400 µl of Matrigel was added into 24-well plates and allowed to stand at 37°C for 30 minutes to gelatinize. Purified SW620^CD133+^ and SW620^CD133−^ cells (500 cells) were seeded on top of each well in 800 µl medium that was changed every 3 days. After 3 weeks, the number of colonies was counted and the colonies were photographed (2.5× objective, NA 0.007). The cells were then recovered from the Matrigel using Cell Recovery Solution (BD, San Jose, CA) for further experiments. The early phase cell proliferation assay used 200 µl of Matrigel in 48-well plates. Cells were seeded in very low cell density (∼200 cells/well), the dispersion of each cell were validated under microscope (DM-IRBE, Leica). After 3 days incubation, the cell numbers of each colony were counted one by one under microscope.

### Immunohistochemical staining

Paraffin-embedded, formalin-fixed tissue sections (5 µm in thickness) were deparaffinized and rehydrated with xylene (Sigma-Aldrich) and gradient alcohol (Sigma-Aldrich, 100%, 90%, 80%). Following antigen retrieval were use Trilogy (Cell Marque, CA) and boiled for 30 min in a pressure cooker. Endogenous peroxidase activity was blocked by incubation with peroxidase block (Dako, EnVision system) for 10 min. Non-specific binding was blocked by incubation with antibody diluent (Dako, CA) for 10 min. Further block endogenous mouse immunoglobulin on mouse tissue with Rodent Block M (Biocare Medical, CA) for 30 min. The slides were incubated with antibodies against human CD133 (Miltenyi Biotec) antibody with antibody diluent at 4°C overnight in a moist chamber. Using diaminobenzidine tetrahydrochloride (DAB) as the substrate. Nuclear counterstaining with hematoxylin. The stained slides were scanned using a Scanscope CS (Aperio).

### Immunofluorescence staining

Cells were fixed with 4% paraformaldehyde (in PBS) for 15 min, blocked with 1% BSA in PBS for 30 min. Incubated with human CD133 (Miltenyi Biotec) antibody in 1% BSA/PBS at 4°C overnight. After PBS washes, secondary antibodies (30 mg/ml goat anti-mouse IgG conjugated with FITC, Jackson ImmunoResearch) were applied at room temperature for 1 h. After HBSS washes, WGA (Invitrogen, alexa fluor 594 conjugate, 1∶200) and hoechst33342 (Invitrogen, 1∶100000) in HBSS were applied at room temperature for 10 min. Following HBSS washes, cells were mounted in mounting medium (Vector Laboratories, Inc., CA) and observed by confocal microscopy (Leica SP5).

### Statistical Analysis

Student's t-test was used to evaluate the statistical significance of the difference in the present study, * indicate that p<0.05, ** indicate that p<0.01, *** indicate that p<0.001.

## Supporting Information

Figure S1 **Comparison of proliferation capacity of SW620^CD133+^ and SW620^CD133−^ cells.** CD133 Expression of SW620 were shown by IF staining for CD133 (green). Nuclear (hoechst33342, blue) and plasma membrane (WGA, red) of cells were co-stained. The arrow indicates spherical type of SW620, while bipolar type was pointed by arrow-head (Figure S1A and S1B). SW620^CD133+^ and SW620^CD133−^ cells were observed to have similar morphologies. MTT assay and CFSE staining revealed no significant difference in cell proliferation ability between the 2 cell subpopulations after 3 and 6 days of culture (Figure S1C). In the CFSE staining results, the x-axis (CD133-APC) shows the CD133^+^ (upper) and CD133^−^ (lower) cell populations, the y-axis (CFSE-FITC) indicates the timing of cell division, the left panel shows the day 0 results, and the right panel shows the day 3 results. The CFSE staining results demonstrate that SW620^CD133+^ and SW620^CD133−^ cells have similar cell-doubling rates (Figure S1D).
(TIF)

Figure S2 **Comparison of drug-resistance capacity of SW620^CD133+^ and SW620^CD133−^ cells.** The results of MTT assay after Taxol (Figure S2A), Cisplatin (Figure S2B), Actinomycin D (Figure S2C), and Camptothecin (Figure S2D) treatment revealed that all treatments inhibit cell proliferation to a similar extent in both subpopulations.
(TIF)

Figure S3 **Histology staining on SW620^CD133+^ and SW620^CD133−^ forming tumor.** H&E staining revealed the tumor morphology were similar between SW620^CD133+^ and SW620^CD133−^ (Figure S3A and S3B). CD133 expressions of two groups were also similar in immunohistochemical staining (Figure S3C and S3D).
(TIF)

Figure S4 **Effect of exposure to hypoxia and ECM coating on viability of SW620^CD133+^ and SW620^CD133−^ cells.** While SW620^CD133+^ and SW620^CD133−^ cells show a similar level of proliferation capacity in a conventional culture system, they show differing levels in a 3D Matrigel culture and *in vivo* in tumors. These differences were further examined by comparing the effect of exposure to hypoxia or ECM coating on cell proliferation by MTT assay. In a less than 1% O_2_ concentration culture chamber (filled diamond in Figure S4A), the proliferation capacity of both subpopulations are significantly lower than in the control (filled circle) after 3 days in culture (SW620^CD133+^: p = 0.007 and SW620^CD133−^: p = 0.003). This hypoxic condition was validated by HIF1-alpha expression (Figure S4B). On an ECM-coated Petri dish (filled triangle), both subpopulations displayed more rapid proliferation than the control (p = 0.024 and 0.022 in SW620^CD133+^ and SW620^CD133−^ respectively). However, no significant differences were observed between the subpopulations regarding their reaction to exposure to hypoxia or ECM coating.
(TIF)
