# Supplementary figures and images for: Response to Stress in Early Tumor Colonization Modulates Switching of CD133-Positive and CD133-Negative Subpopulations in a Human Metastatic Colon Cancer Cell Line, SW620
Source: PLoS One. 2013 Apr 5;8(4):e61133. doi: 10.1371/journal.pone.0061133 (PMC3618272; doi:10.1371/journal.pone.0061133)

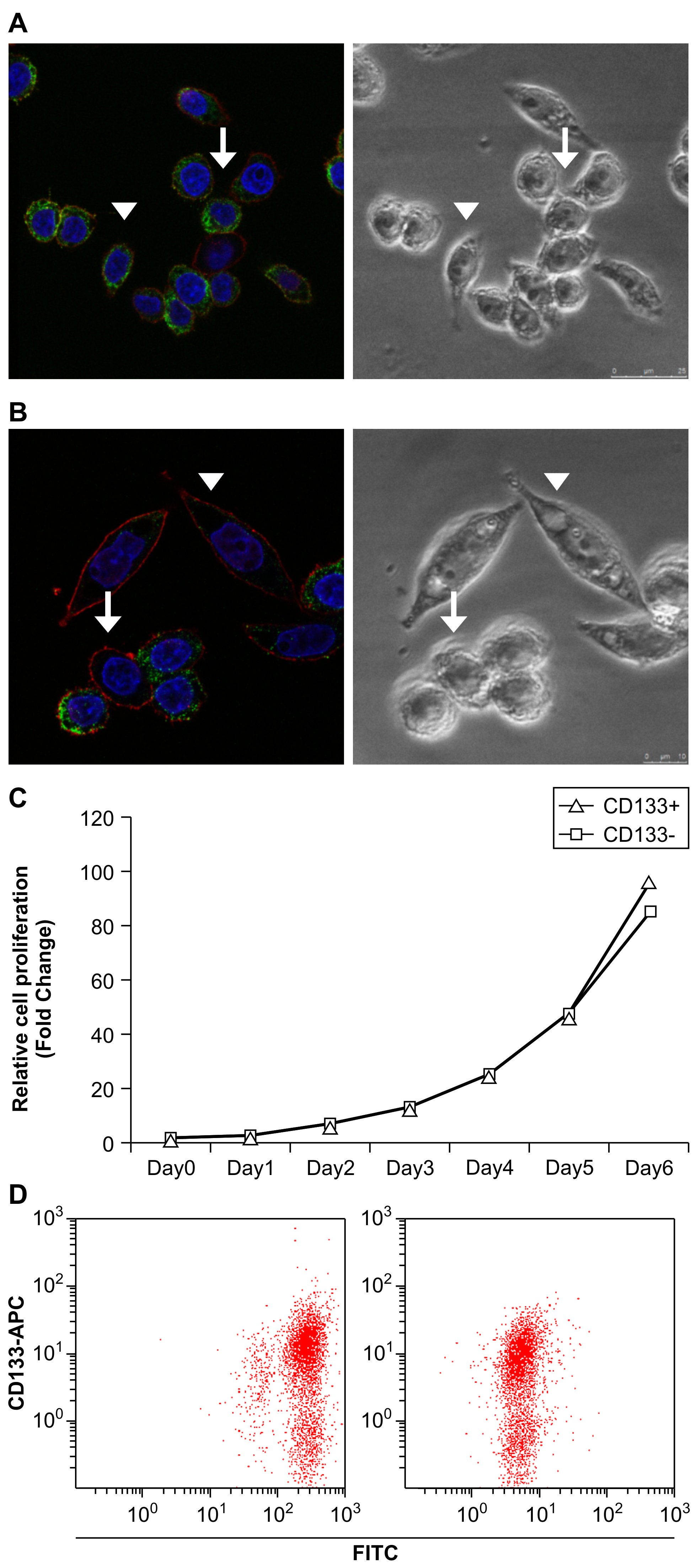

Supplement: Figure S1 — Comparison of proliferation capacity of SW620CD133+ and SW620CD133− cells. CD133 Expression of SW620 were shown by IF staining for CD133 (green). Nuclear (hoechst33342, blue) and plasma membrane (WGA, red) of cells were co-stained. The arrow indicates spherical type of SW620, while bipolar type was pointed by arrow-head (Figure S1A and S1B). SW620CD133+ and SW620CD133− cells were observed to have similar morphologies. MTT assay and CFSE staining revealed no significant difference in cell proliferation ability between the 2 cell subpopulations after 3 and 6 days of culture (Figure S1C). In the CFSE staining results, the x-axis (CD133-APC) shows the CD133+ (upper) and CD133− (lower) cell populations, the y-axis (CFSE-FITC) indicates the timing of cell division, the left panel shows the day 0 results, and the right panel shows the day 3 results. The CFSE staining results demonstrate that SW620CD133+ and SW620CD133− cells have similar cell-doubling rates (Figure S1D). (TIF) [file pone.0061133.s001.tif]

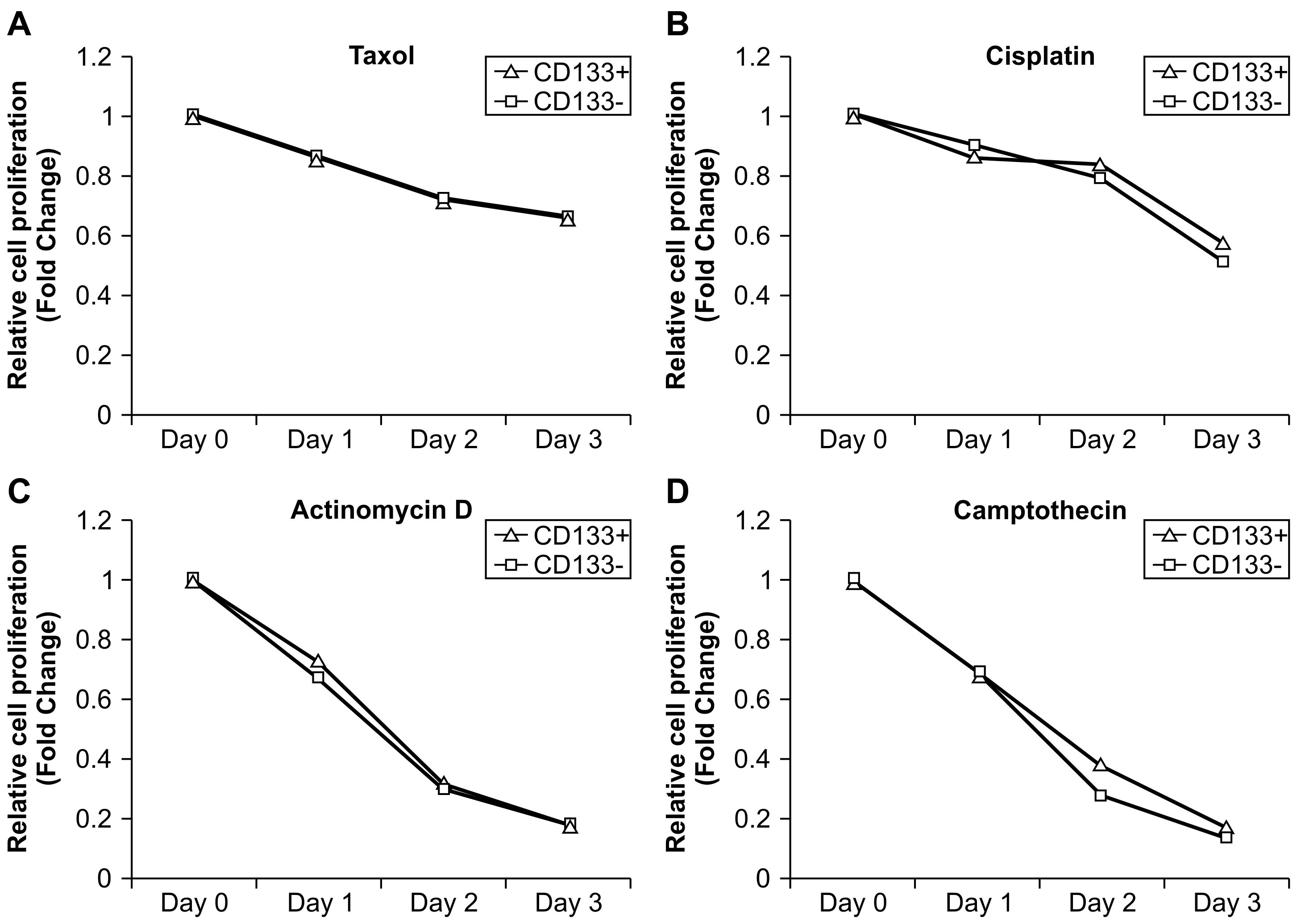

Supplement: Figure S2 — Comparison of drug-resistance capacity of SW620CD133+ and SW620CD133− cells. The results of MTT assay after Taxol (Figure S2A), Cisplatin (Figure S2B), Actinomycin D (Figure S2C), and Camptothecin (Figure S2D) treatment revealed that all treatments inhibit cell proliferation to a similar extent in both subpopulations. (TIF) [file pone.0061133.s002.tif]

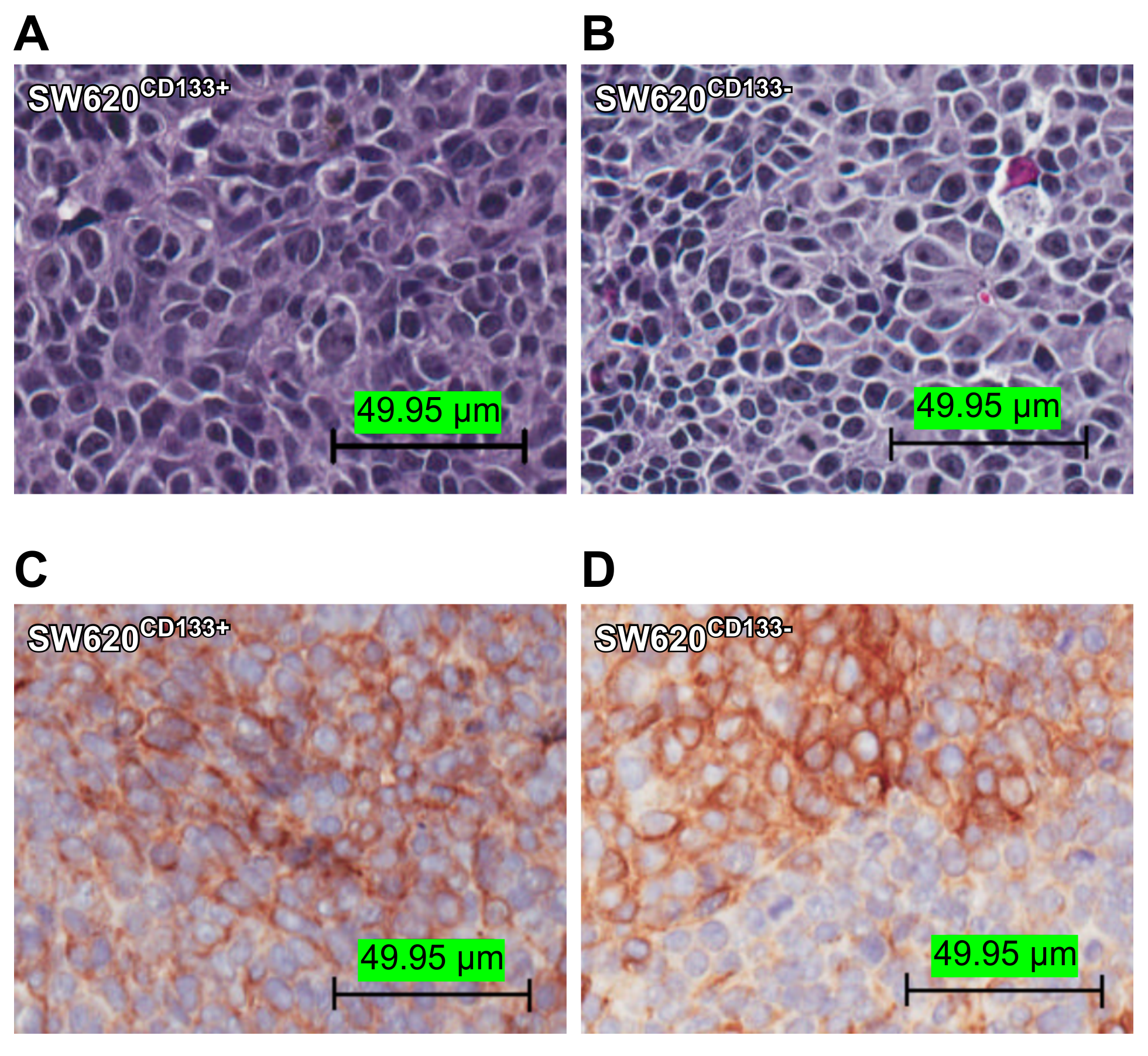

Supplement: Figure S3 — Histology staining on SW620CD133+ and SW620CD133− forming tumor. H&E staining revealed the tumor morphology were similar between SW620CD133+ and SW620CD133− (Figure S3A and S3B). CD133 expressions of two groups were also similar in immunohistochemical staining (Figure S3C and S3D). (TIF) [file pone.0061133.s003.tif]

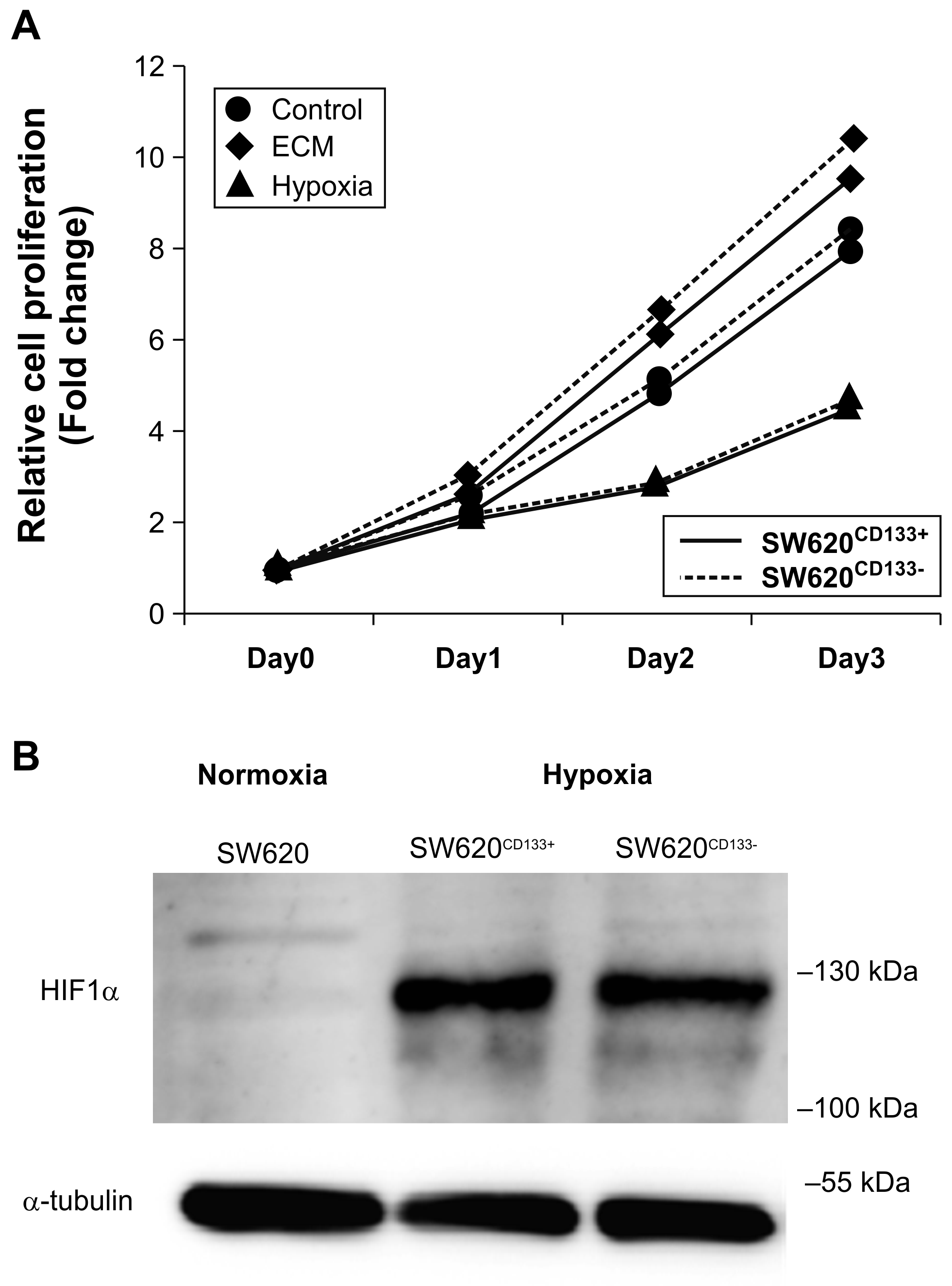

Supplement: Figure S4 — Effect of exposure to hypoxia and ECM coating on viability of SW620CD133+ and SW620CD133− cells. While SW620CD133+ and SW620CD133− cells show a similar level of proliferation capacity in a conventional culture system, they show differing levels in a 3D Matrigel culture and in vivo in tumors. These differences were further examined by comparing the effect of exposure to hypoxia or ECM coating on cell proliferation by MTT assay. In a less than 1% O2 concentration culture chamber (filled diamond in Figure S4A), the proliferation capacity of both subpopulations are significantly lower than in the control (filled circle) after 3 days in culture (SW620CD133+: p = 0.007 and SW620CD133−: p = 0.003). This hypoxic condition was validated by HIF1-alpha expression (Figure S4B). On an ECM-coated Petri dish (filled triangle), both subpopulations displayed more rapid proliferation than the control (p = 0.024 and 0.022 in SW620CD133+ and SW620CD133− respectively). However, no significant differences were observed between the subpopulations regarding their reaction to exposure to hypoxia or ECM coating. (TIF) [file pone.0061133.s004.tif]
